# Poster Session I - A179 SYSTEMATIC REVIEW OF GASTRIC POLYP OUTLET OBSTRUCTION: DIAGNOSIS AND MANAGEMENT

**DOI:** 10.1093/jcag/gwaf042.179

**Published:** 2026-02-13

**Authors:** S Woo, H Huang, B Nguyen

**Affiliations:** Undergraduate Medicine, The University of British Columbia Faculty of Medicine, Vancouver, BC, Canada; Undergraduate Medicine, The University of British Columbia Faculty of Medicine, Vancouver, BC, Canada; Undergraduate Medicine, The University of British Columbia Faculty of Medicine, Vancouver, BC, Canada

## Abstract

**Background:**

Gastric outlet obstruction (GOO) presents with nausea, vomiting, weight loss, early satiety, and abdominal distension due to impaired gastric emptying. While most gastric polyps are asymptomatic, larger polyps may prolapse through the pylorus and cause GOO in a “ball-valve” manner and may undergo malignant transformation. Current evidence is limited to case reports, case series, and reviews. This systematic review synthesizes primary data on presentation, diagnosis, management, and outcomes of GOO secondary to gastric polyps.

**Aims:**

To summarize the evidence on symptomatology, diagnostic approaches, and treatment of gastric polyp–related GOO.

**Methods:**

A PRISMA-based systematic review of MEDLINE, Embase, Scopus, and Web of Science was conducted. Descriptive statistics were used for demographics and treatment outcomes.

**Results:**

Of 408 articles, 56 (52 case reports, 4 case series) published up to April 2025 were included. Median age at presentation was 47 years (range 2 days–89 years) with female predominance. Common symptoms included epigastric/abdominal pain, nausea, and vomiting. Polyps were usually in the antrum with a median size of 4 cm. Upper endoscopy was diagnostic in 91.8%, often revealing a pedunculated polyp prolapsing into the duodenum. CT was used in 34% as adjunct imaging. Endoscopic resection was performed in 24/61 cases (39.3%) and was curative in all. Surgical management was required in 27/61 (44.3%), especially for large or sessile lesions. Symptom resolution occurred in 58/61 cases (95%).

**Conclusions:**

In patients with weight loss, vomiting, and anemia, malignancy should first be suspected due to overlapping symptoms. GOO is a clinical diagnosis, and benign polypoidal causes should be considered once malignancy is rule out. Given malignant potential, polypectomy is first-line, with endoscopic or surgical approach based on polyp size and extent. Both methods show excellent outcomes and no procedure-related mortality. Hereditary polyposis syndromes should be considered in patients with personal or family history. Management should be individualized based on presentation, comorbidities, histology, and extent of polyposis. Further long-term studies are needed to assess recurrence after polypectomy.

**Funding Agencies:**

None

**A179 Table 1:** Polyp size, response to treatment and recurrence

Treatment approach	n/N (%)	Median polyp size (cm)	Recurrence n/N (%)
Endoscopic resection	24/61 (39.3%)	3.5	0/24
Surgical resection	27/61 (44.3%)	5.05	0/27
Endoscopic + adjunctive therapy	5/61 (0.08%)	3	1/5
Surgery + adjunctive therapy	1/61 (0.02%)	4	1/1
Surgical after previous non-surgical attempts	3/61 (0.05%)	6.75	0/3
Medical only	1/61 (0.02%)	Unreported	1/1

